# Six SIGMA evaluation of 17 biochemistry parameters using bias calculated from internal quality control and external quality assurance data

**DOI:** 10.5937/jomb0-43052

**Published:** 2024-01-25

**Authors:** Tülay Çevlik, Goncagül Haklar

**Affiliations:** 1 Marmara University Pendik E&R Hospital, Biochemistry Laboratory, Istanbul, Turkey; 2 Marmara University, School of Medicine, Department of Biochemistry, Istanbul, Turkey

**Keywords:** Six Sigma method, quality goal index, quality management, imprecision, bias, Six Sigma metoda, indeks kvaliteta, upravljanje kvalitetom, nepreciznost, pristranost

## Abstract

**Background:**

Six Sigma is a popular quality management system that enables continuous monitoring and improvement of analytical performance in the clinical laboratory. We aimed to calculate sigma metrics and quality goal index (QGI) for 17 biochemical analytes and compare the use of bias from internal quality control (IQC) and external quality assurance (EQA) data in the calculation of sigma metrics.

**Methods:**

This retrospective study was conducted in Marmara University Pendik E&R Hospital Biochemistry Laboratory. Sigma metrics calculation was performed as (TEa-bias)/CV). CV was calculated from IQC data from June 2018 - February 2019. EQA bias was calculated as the mean of % deviation from the peer group means in the last seven surveys, and IQC bias was calculated as (laboratory control result mean-manufacturer control mean)/ manufacturer control mean) x100. In parameters where sigma metrics were <5; QGI=bias/1.5 CV) score of <0.8 indicated imprecision, >1.2 pointed inaccuracy, and 0.8-1.2 showed both imprecision and inaccuracy.

**Results:**

Creatine kinase (both levels), iron and magnesium (pathologic levels) showed an ideal performance with ≥6 sigma level for both bias determinations. Eight of the 17 parameters had different sigma levels when we compared sigma values calculated from EQA and IQC derived bias% while the rest were grouped at the same levels.

**Conclusions:**

Sigma metrics is a good quality tool to assess a laboratory's analytical performance and facilitate the comparison of the assay performances in the same manner across multiple systems. However, we might need to design a tight internal quality control protocol for analytes showing poor assay performance.

## Abstract (sr)

**Uvod:**

Six Sigma je popularni sistem za upravljanje kvalitetom koji omogućava kontinuirano praćenje i poboljšanje analitičkih performansi u kliničkim laboratorijama. Cilj nam je bio da se izračuna sigma metrika i indeks kvaliteta (Quality Goal Index - QGI) za 17 biohemijskih analita i uporedi upotreba pristranosti iz podataka interne kontrole kvaliteta (Internal Quality Control - IQC) i eksterne kon trole kvaliteta (External Quality Assurance - EQA) u izraču navanju sigma metrike.

**Metode:**

Ova retrospektivna studija je sprovedena u Biohemijskoj laboratoriji Bolnice Marmara University Pendik E&R. Izračunavanje sigma metrike je izvršeno kao (TEa-pristranost)/CV). CV je izračunat iz IQC podataka od juna 2018. do februara 2019. EQA pristranost je izračunata kao srednja vrednost % odstupanja od srednjih vrednosti grupe vršnjaka u poslednjih sedam anketa, a IQC pristranost je izračunata kao (srednja vrednost rezultata laboratorijske kontrole-srednja vrednost kontrolne materije proizvođača)/srednja vrednost kontrolne materije pro izvođača) x 100. Za parametre kod kojih je sigma metrika bila <5; QGI= pristrasnost/ 1,5 CV), rezultat <0,8 je uka zivao na nepreciznost, >1,2 na netačnost, a rezultat u rasponu od 0,8 do 1,2 je ukazivao na nepreciznost i netačnost.

**Rezultati:**

Kreatin kinaza (oba nivoa), gvožđe i magnezijum (patološki nivoi) su pokazali idealne performanse sa ≥6 sigma nivoima za obe određene pristranosti. Osam od 17 parametara je imalo različite sigma nivoe kada smo uporedili sigma vrednosti izračunate iz EQA i IQC pristranosti, dok su ostali bili grupisani na istim nivoima.

**Zaključak:**

Sigma metrika je dobar alat za ocenu analitičkih performansi laboratorije i olakšava upoređivanje performansi analize na isti način na više sistema. Međutim, možda će biti potrebno da se ponovo osmisli stroži protokol interne kontrole kvaliteta za analite koji pokazuju loše analitičke performanse.

## Introduction

Clinical laboratories are constantly making great efforts to produce more efficient and reliable results. Quality control plays a vital role in the laboratory’s ability to produce accurate test results, and it has been a component of laboratory medicine for more than 50 years. While the first quality control methods were designed to detect errors visually, in the 1930s, Shewhart and his colleagues pioneered statistical analysis in the control process. Then, Levey and Jennings [1] [2] charts, Westgard multiple rules, and Sigma metric are successfully added to quality control management. Using statistical data to monitor the process has enabled the laboratorians to reduce the possibility of producing inappropriate results.

More than 70% of clinical decisions are based on laboratory results, and most clinicians believe that laboratories produce test results with zero errors. However, there is no zero-fault process [3]. Errors can occur at any time during the testing process while controlling and monitoring is the task of the laboratory specialists. It is crucial to minimise the error rate regarding patient safety [1] [4].

Depending upon the time of presentation, laboratory errors are observed in preanalytical (46–68%), analytical (7–13%), and post-analytical (18.5–47%) phases, and performing quality studies for all phases is of great importance for the reliability of the results. In the analytical phase, quality indicators encompass the internal and external quality control results. While the quality indicator for the preanalytical phase is the rejected sample rate, in the post-analytic phase, they are the rate of results that were not given on time and the rate of unreported panic value. Another approach is to use the Six Sigma concept to evaluate preanalytical, analytical, and post-analytic phases [5] [6].

Six Sigma is a quality management tool used for monitoring and improving the performance of analytical processes in the clinical laboratory. It is not just a tool to describe process performance but also detects errors and eliminates their causes as much as possible to reach the zero error goal [7] [8]. The Six Sigma scale ranges from 0 to 6, and as the Sigma value increases, the performance of the test increases. While a sigma value of 6 in a process indicates that the performance of the test is excellent, the lowest sigma value for acceptable performance is 3. When the analysis performance 5 sigma, the quality goal index (QGI) should be calculated to determine the cause of poor performance. The two most important concepts that drive analytic quality management are accuracy and precision. QGI determines the problems of inaccuracy and imprecision for the analytical processes concerning their quality goals [9].

In this study, we aimed to calculate sigma metrics and quality goal index (QGI) for 17 biochemical analytes and compare the use of bias from internal quality control (IQC) and external quality assurance (EQA) data in the calculation of sigma metrics.

## Materials and methods

The study was retrospectively conducted in Marmara University Pendik E&R Hospital Biochemistry Laboratory. A total of 17 parameters, namely albumin, alkaline phosphatase (ALP), alanine aminotransferase (ALT), amylase, aspartate aminotransferase (AST), creatine kinase (CK), iron, inorganic phosphate, glucose, calcium (Ca), creatinine, magnesium (Mg), total bilirubin, total cholesterol, total protein, triglycerides, and uric acid were enrolled. The analyses were done by an AU5800 biochemistry analyser (Beckman Coulter, USA), and data were collected over a 7-month period (01/June/2018–28/February/2019). According to our quality control schedule, we ran 2 levels of IQC samples (Bio-Rad, USA) every day and one level of EQA sample (RIQAS, UK) per month, either normal (N) or pathological (P) as defined by the external agency. The sigma metrics were estimated according to the formula below using bias, CV, and total allowable error (TEa) [3].

Sigma = (TEa-bias)/CV.

The coefficient of variation (CV) was calculated for each IQC level separately.

CV (%) = (SD/mean) x100

The bias % for each parameter was calculated from both IQC and EQA data.

Bias (%) = [(our IQC or EQA result – Beckman Coulter IQC mean, or peer group mean of EQA)/Beckman Coulter IQC mean, or peer group mean of EQA] x100

Total allowable error (TEa) values were decided according to Clinical Laboratory Improvement Amendments Specifications (CLIA’88).

Analytes were classified according to their performance as follows: ≥6 sigma level: excellent performance, <6–≥5 sigma: very good or perfect, <5–≥4 sigma: good, <4–≥3 sigma: medium, 3 sigma: poor performance.

Statistical Package for the Social Sciences (SPSS Inc.; Chicago, IL, USA) software was used to analyse the sigma values obtained from internal and external quality control data. We checked for normality using the Kolmogorov-Smirnov test. To compare the mean difference between the two groups, we conducted a paired t-test. A group with less than three sigma values was considered unsuccessful, while more than three were deemed successful. The Mc Nemar test for paired samples was used to compare the sigma values.

Quality goal index (QGI) values were calculated to understand the reason behind lower performances according to sigma metrics [6].

QGI = Bias /1.5 CV

For analytes that fall short of Six Sigma quality, a QGI ratio of <0.8 indicates imprecision, a ratio >1.2 indicates inaccuracy, and a QGI ratio between 0.8–1.2 indicates both imprecision and inaccuracy. CV, bias, Sigma and QGI values were calculated with Microsoft Excel Software.

## Results

Average CV (%) values of 17 biochemistry parameters calculated from IQC samples over 7 months range between 3.05–5.87 for level 1 and 2.4–4.72 for level 2. Bias (%) values were calculated from IQC and EQA for the same period (Table 1).

**Table 1:** Average CV (%) values of level 1 and 2 IQC samples, bias (%) values calculated from IQC and EQA samples, and TEa values obtained from CLIA'88 for 17 biochemistry parameters.

Parameter	CV (%)		IQC<br>bias (%)	EQA<br>bias (%)	TEa<br>(CLIA’88)
	Level 1	Level 2			
Albumin	3.48	2.88	0.60	1.78	10
ALP	5.87	4.64	3.41	5.67	30
ALT	4.45	2.81	2.26	5.88	20
Amylase	3.57	2.66	14.10	5.32	30
AST	3.75	3.39	4.38	3.82	20
CK	3.3	2.4	2.35	4.81	30
Iron	3.54	2.7	0.51	3.5	20
Inorganic phosphate	4.94	4.72	1.08	2.75	10
Glucose	3.64	3.19	1.78	1.20	10
Calcium	4.68	3.2	1.81	1.34	11
Creatinine	4.44	3.65	6.32	5.42	15
Magnesium	4.24	3.17	0.28	1.97	25
Total bilirubin	4.5	4.24	4.57	2.11	20
Total cholesterol	3.34	3.03	0.58	1.25	10
Total protein	3.80	2.82	0.94	0.68	10
Triglyceride	4.56	4.58	2.31	4.88	25
Uric acid	3.05	2.64	1.45	2.30	17

The sigma and QGI values calculated using the bias obtained from EQA data (either N or P samples) were summarised in Table 2. Among the 17 analytes, the performances of amylase (N and P), CK (N and P), iron (P), and magnesium (P) were excellent with >6 sigma level. ALP (P), ALT (P), magnesium (N), and uric acid (P) showed very good sigma performance, while the performances of ALP (N), AST (N and P), iron (N), triglyceride (N and P), uric acid (N) were good with <5–≥4 sigma level. On the other hand, the performances of ALT (N), calcium (P), total bilirubin (N), and total protein (P) were medium with <4–≥3, sigma level while albumin (N and P), inorganic phosphate (N and P), glucose (N and P), calcium (N), creatinine (N and P), total cholesterol (N and P), and total protein (N) had poor performances (sigma metrics was less than 3).

**Table 2:** Sigma metrics and QGI for parameters with Sigma <5 calculated using bias values derived from EQA data (*imprecision, **imprecision and inaccuracy).

Parameter	Sigma EQA		QGI EQA		Problem
	Normal	Pathologic	Normal	Pathologic	
Albumin	2.36	2.85	0.34*	0.41*	Imprecision (N and P)
ALP	4.14	5.24	0.64*		Imprecision (N)
ALT	3.17	5.02	0.88**		Imprecision and inaccuracy (N)
Amylase	6.91	9.28			
AST	4.31	4.77	0.68*	0.75*	Imprecision (N and P)
CK	7.63	10.50			
Iron	4.66	6.11	0.66*		Imprecision (N)
Inorganic phosphate	1.47	1.54	0.37*	0.39*	Imprecision (N and P)
Glucose	2.42	2.76	0.22*	0.25*	Imprecision (N and P)
Calcium	2.06	3.02	0.19*	0.28*	Imprecision (N and P)
Creatinine	2.16	2.62	0.81**	0.99**	Imprecision and inaccuracy<br>(N and P)
Magnesium	5.43	7.26			
Total Bilirubin	3.98	4.22	0.31*	0.33*	Imprecision (N and P)
Total cholesterol	2.62	2.89	0.25*	0.28*	Imprecision (N and P)
Total protein	2.45	3.30	0.12*	0.16*	Imprecision (N and P)
Triglyceride	4.41	4.39	0.71*	0.71*	Imprecision (N and P)
Uric acid	4.82	5.57	0.50*		Imprecision (N)

The QGI values were calculated for parameters which had analytic performance <5 sigma and the leading causes of poor performance were determined accordingly. The performance of 7 parameters (ALP, ALT, amylase, CK, iron, magnesium, and uric acid) at one or more EQA levels was very good or excellent. Twelve parameters (albumin, ALP, AST, iron, inorganic phosphate, glucose, calcium, total bilirubin, total cholesterol, total protein, triglyceride, and uric acid) exhibited precision problems at one or more EQA levels. In comparison, two parameters (ALT and creatinine) showed that there were accuracy and precision problems at one or more EQA levels (Table 2).

The sigma and QGI values which were determined using the bias calculated from the IQC data analysed daily between June 2018 and February 2019, were given in Table 3.

**Table 3:** Sigma metrics and QGI for parameters with Sigma <5 calculated using bias values derived from IQC data (* imprecision, ** inaccuracy, ***imprecision and inaccuracy).

Parameter	Sigma IQC		QGI IQC		Problem
	Level 1	Level 2	Level 1	Level 2	
Albumin	2.70	3.26	0.11*	0.14*	Imprecision (level 1and 2)
ALP	4.53	5.73	0.39*		Imprecision (level 1)
ALT	3.98	6.30	0.34*		Imprecision (level 1)
Amylase	4.45	5.98	2.63**		Inaccuracy (level 1)
AST	4.16	4.60	0.78*	0.87***	Imprecision (level 1). imprecision and<br>inaccuracy (level 2)
CK	8.39	11.54			
Iron	5.51	7.22			
Inorganic phosphate	1.80	1.89	0.15*	0.16*	Imprecision (level 1 and 2)
Glucose	2.25	2.57	0.33*	0.38*	Imprecision (level 1 and 2)
Calcium	1.97	2.88	0.26*	0.38*	Imprecision (level 1 and 2)
Creatinine	1.96	2.38	0.95***	1.15***	Imprecision and inaccuracy<br>(level 1 and 2)
Magnesium	5.83	7.79			
Total Bilirubin	3.42	3.63	0.68*	0.72*	Imprecision (level 1 and 2)
Total cholesterol	2.81	3.10	0.12*	0.13*	Imprecision (level 1 and 2)
Total protein	2.39	3.23	0.16*	0.21*	Imprecision (level 1 and 2)
Triglyceride	4.98	4.96	0.34*	0.33*	Imprecision (level 1 and 2)
Uric acid	5.08	5.87			

ALT (level 2), CK (level 1 and level 2), iron (level 2), and magnesium (level 2) tests have shown ideal performance with ≥6 sigma. ALP (level 2), amylase (level 2), iron (level 1), magnesium (level 1), and uric acid (levels 1 and 2) showed <6–≥5 sigma performance. ALP (level 1), amylase (level 1), AST (level 1 and 2), and triglyceride (level 1 and 2) parameters showed good performance with <5–≥4 sigma. On the other hand, albumin (level 2), ALT (level 1), total bilirubin (level 1 and level 2), total cholesterol (level 2), and total protein (level 2) parameters have shown medium performance with <4–≥3 sigma. The parameters below sigma level 3 were albumin (level 1), inorganic phosphate (levels 1 and 2), glucose (levels 1 and 2), calcium (levels 1 and 2), creatinine (levels 1 and 2), total cholesterol (level 1), and total protein (level 1).

The QGI values parameters with analysis performance <5 sigma were also given in Table 3. These values were calculated using 2 levels of IQC bias data, and the leading causes of poor performancewere determined accordingly. The performance of seven parameters (ALP, ALT, amylase, CK, iron, magnesium, and uric acid) at one or more IQC levels was either very good or excellent. Eleven parameters (Albumin, ALP, ALT, AST, inorganic phosphate, glucose, calcium, total bilirubin, total cholesterol, total protein, triglyceride) exhibited precision problems, two parameters (AST and creatinine) showed that there were problems with accuracy and precision. In contrast, one parameter (amylase) showed accuracy problems at one or more IQC levels.

The mean and standard deviation of internal quality control sigma values were 4.38 ±2.17, while external quality control sigma values had a mean and SD of 4.30±2.13, respectively. However, the paired t-test showed no statistically significant difference (P=0.547) between the two groups. The performance evaluation for sigma values showed no statistically significant difference between the two paired groups (McNemar’s test, P=1.000).

Westgard has determined the preference of IQC rules according to sigma levels (10). We have selected the rules according to sigma levels calculated using EQA and ICQ bias data (Table 4).

**Table 4:** Quality control procedures selected for parameters.

Parameters with sigma values<br>calculated according to EQA	Parameters with sigma values<br>calculated according to IQC	Sigma<br>value	Control rule	Run
ALT (N), calcium (P),<br>total bilirubin (N), total protein (P)	Albumin (level 2), ALT (level 1),<br>total bilirubin (level 1 and level 2), total<br>cholesterol (level 2), total protein (level 2)	3-<4<br>sigma	1_3s_/2_2s_/R_4s_/4_1s_/8_x_	R=1,<br>N=4
ALP (N and P), ALT (P),<br>magnesium (N), AST (N and P),<br>iron (N) triglyceride (N and P),<br>uric acid (N and P)	ALP (level 1 and 2), AST (level 1 and 2),<br>triglyceride (level 1 and 2), amylase (level 2),<br>iron (level 1), magnesium (level 1),<br>uric acid (level 1 and 2)	4-6<br>sigma	12.5s	R=1,<br>N=2
Amylase (N and P), CK (N and P),<br>iron (N), magnesium (P)	ALT (level 2), CK (level 1 and 2), iron<br>(level 2), magnesium (level 2)	≥6<br>sigma	13s	R=1,<br>N=2

## Discussion

Sigma methodology is one of the critical process improvement tools used in evaluating analytical performance, revealing errors in accuracy and precision together. It is based on calculations emphasising the use of quantitative techniques to measure the actual performance of the process [10]. Detecting and eliminating errors increases the testing process’s quality. Sigma methodology is used for quantitative comparison of laboratories and methods, allowing the laboratory to determine its quality control strategy.

In this study, we have evaluated the analytical performance of 17 biochemistry parameters, namely albumin, ALP, ALT, amylase, AST, CK, iron, inorganic phosphate, glucose, calcium, creatinine, magnesium, total bilirubin, total cholesterol, total protein, triglycerides, uric acid. We have calculated the sigma values of these tests using the bias values derived from IQC and EQA data and selected Westgard quality control rules accordingly. We have also calculated each parameter’s quality goal index (QGI) to determine whether the problem in tests with a sigma value <6 was imprecision or inaccuracy [8] [11] [12].

We have calculated bias % using EQA or IQC values, which both had 2 levels, and we have obtained 68 sigma values for 17 biochemistry parameters. Eight parameters (albumin, ALT, amylase, iron, calcium, total bilirubin, total cholesterol, and uric acid) had different sigma levels when we compared sigma values calculated from EQA and IQC-derived bias %, while the rest of the parameters were grouped at same levels. IQC-derived sigma values of albumin (level 2), ALT (level 2), iron (level 1), total cholesterol (level 2), and uric acid (level 1) were higher than EQA-derived counterparts. On the other hand, amylase (levels 1 and 2), iron (level 1), Ca (level 2), and total bilirubin (level 2) had IQC-derived sigma values lower than their EQA-derived counterparts.

When QGI was calculated using EQA-derived bias, albumin (N and P), ALP (N), AST (N and P), iron (N), inorganic phosphate (N and P), glucose (N and P), Ca (N and P), total bilirubin (N and P), total cholesterol (N and P), total protein (N and P), triglycerides (N and P), and uric acid (N and P), parameters were below 0.8. We interpreted this as an error in imprecision, which is an expression of random error. Random errors cannot be predicted in magnitude and direction; therefore, they can be positive or negative and occur with repeated measurements. These errors occur due to unstable electrical supply, unstable temperature and incubation conditions, bubbles in reagents and reagent lines, individual operator variation in pipetting, timing, etc. Therefore, these conditions should be reviewed in tests with a QGI below 0.8. QGI values of ALT (level 1) and creatinine (level 1 and level 2) were calculated between 0.8 and 1.2. In these tests, the source of the problem was determined as an error in both precision and accuracy which showed the presence of both random and systematic errors. Systematic errors are always in the same direction, either positive or negative. Therefore, to increase performance in these tests, situations that may cause both random and systematic errors should be reviewed and controlled [13].

Among 17 parameters, ALT (level 2), CK (level 1 and level 2), iron (level 2), and Mg (level 2) had a sigma value of six or above, and the daily IQC evaluation can be done with one level of IQC sample by using 1_3s_ Westgard rule alone. ALP (levels 1 and 2), AST (levels 1 and 2), amylase (level 2), Mg (level 1), iron (level 1), triglyceride (levels 1 and 2), uric acid (levels 1 and 2) with sigma values between 4 and 6 the performance of the tests can be evaluated with two levels of control once daily with 1_2.5s_ Westgard rule alone. Albumin (level 2), ALT (level 1), total bilirubin (level 1), total cholesterol (level 2), and total protein (level 2) parameters with sigma values between 3 and 4 the use of two levels of controls twice daily with 1_3s_/2_2s_/R_4s_/4_1s_/8_x_ Westgard’s multirule is necessary.

We have performed root cause analysis (RCA) for parameters with sigma values below 3 to review and control the causes that lead to poor performance. RCA is a systematic process to develop corrective actions by identifying the causes of undesirable events and the factors that cause changes in performance [14] [15]. Fishbone diagrams should be prepared and evaluated for possible error causes [16]. In the RCA analysis of tests with low sigma values (<3), the number of operators, performances of devices and methods, and environmental factors must be questioned. The laboratory should consider using alternative methods [17].

Numerous studies were done, and several investigators reported different sigma metrics. As an example, Singh et al. [15] have calculated sigma values of 3.9 and 3.5 for total protein (levels 1 and 2), while the values were 3.0 and 3.2 in the study of Kumar et al. [6]. Differences in sigma values between our research and other studies may arise due to the type of analysers, quality control materials, reagents, or differences in calibration. In addition, the different sources from which the total allowable error values are taken may cause the sigma values to differ between studies.

With Sigma methodology, quality control processes that need to be implemented are selected, contributing to improving analytical performance. When calculating sigma values, both IQC and EQA data are valuable in evaluating the analytical stage, and we have seen minimal differences between the sigma calculations based on them. We concluded that bias % could be calculated from IQC data when external quality assurance data were unavailable. We must prefer test-specific quality control rules since the sigma values are different for different tests. It should be remembered that Sigma evaluation processes can significantly contribute to the laboratory in terms of total quality.

## Dodatak

### List of Abbreviations

QGI, quality goal index;<br>IQC, internal quality control;<br>EQA, external quality assurance;<br>tea, total allowable error;<br>CV, coefficient of variation;<br>ALP, alkaline phosphatase;<br>ALT, alanine aminotransferase;<br>AST, aspartate aminotransferase;<br>CK, creatine kinase;<br>Ca, calcium;<br>Mg, magnesium;<br>N, normal;<br>P, pathologic.

### Funding

This research received no specific grant from public, commercial, or not-for-profit funding agencies.

### Ethics declarations

Ethics approval is not required as the data is selected from internal and external quality control.

### Contributions

GH and TÇ researched the literature and conceived the study. GH and TÇ were involved in protocol development, data recruitment, and analysis. TÇ wrote the first draft of the manuscript. All authors have read and approved the final version.

### Acknowledgements

We would like to thank the healthcare professionals working in the Biochemistry laboratory of Marmara University Pendik Training and Research Hospital.

### Conflict of interest statement

All the authors declare that they have no conflict of interest in this work.
